# Supplementary material for: Reconfigurable two-dimensional optoelectronic devices enabled by local ferroelectric polarization
Source: Nat Commun. 2019 Jul 26;10:3331. doi: 10.1038/s41467-019-11328-0 (PMC6659647; doi:10.1038/s41467-019-11328-0)
Supplement: Supplementary file 1 — Supplementary information [file 41467_2019_11328_MOESM1_ESM.pdf]

Supplementary Information

Reconfigurable Two-Dimensional Optoelectronic Devices Enabled by Local Ferroelectric Polarization

Lv *et al.*

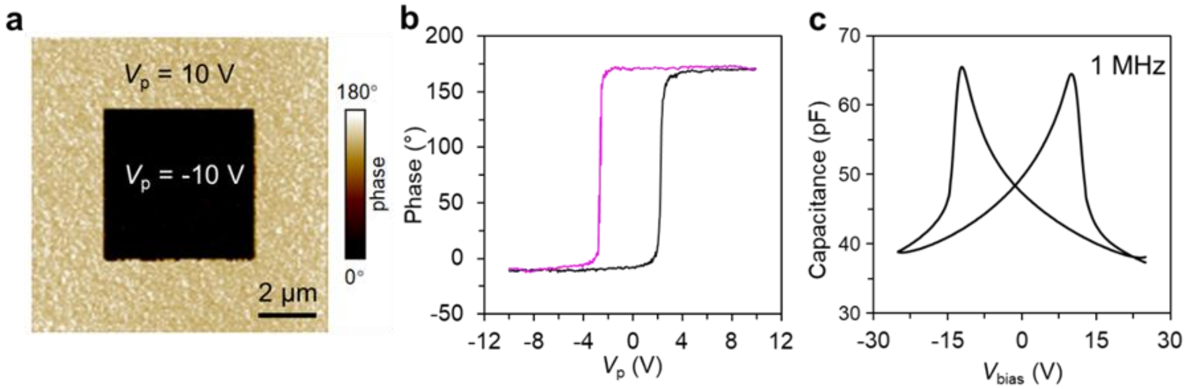

Supplementary Figure 1. Ferroelectric switching characteristic of P(VDF-TrFE) copolymer. **a** Piezoelectric force microscope (PFM) phase image of selectively polarized P(VDF-TrFE) thin film using AFM system. **b** The hysteresis loop when ramping the poling voltage measured at a single point. **c** Capacitance-voltage characteristic of the sandwiched 160 nm P(VDF-TrFE) thin film at 1 MHz.

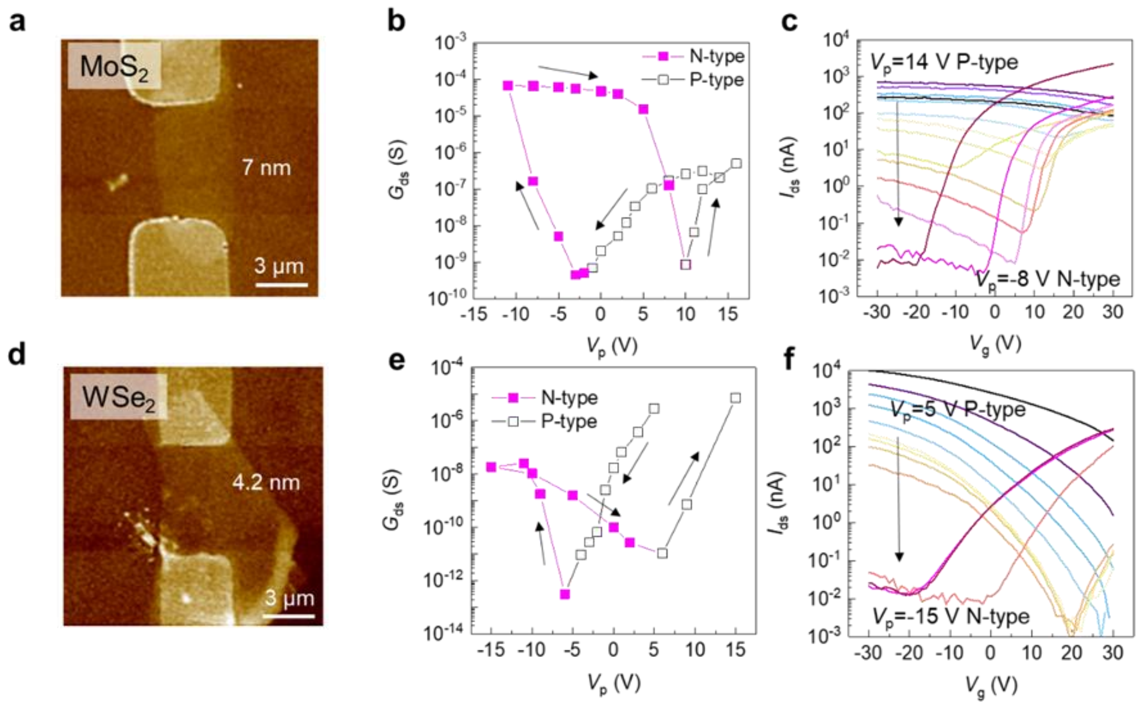

Supplementary Figure 2. Ferroelectric (FE) polarization tuned conductance in MoS_2 and WSe_2 devices. **a**, **d** Atomic force microscope (AFM) image of measured MoS_2 and WSe_2 devices. **b**, **e** FE polarization tuned conductance in MoS_2 and WSe_2 channel, and **c**, **f** their corresponding transfer curves after each polarization state when sweeping V_p in the negative direction (from 14 to -8V in **c**, and 5 to -15V in **f**).

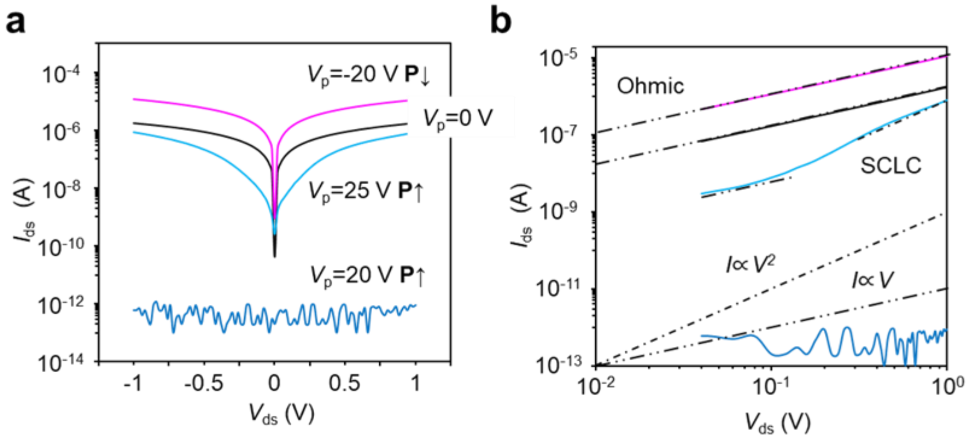

Supplementary Figure 3 *I-V* characteristic of MoS_2 channel under different ferroelectric polarization states. **a** *I-V* curves in linear scale for the same MoS_2 device after applying different poling voltages of V_p= 0 V, -20 V, 25 V, 20 V. **b** displays the plot in log scale, revealing the space charge limited current (SCLC) in p-doped MoS_2 channel under large bias, which indicates the presence of trap filling induced space charges in channel.

Supplementary Note 1. Carrier concentration tuned by FE polarization.

Here, we further attempt to extract the p/n doping concentration under FE polarization from the measured transfer curves of MoS_2 and WSe_2 by Si back-gate after each FE polarization. This is achived with n, or $p=\sigma/\mu e$ calculated from the extracted conductance (σ) at V_{bg}=0 and the estimated carrier mobility from $\mu = (L/W)\, V_{ds}^{-1}\, C_{gate}^{-1}\, (dI_{ds}/dV_g)$, where C_{gate} is the gate coupling capacitance with 2D channel. Correct mobility evaluation is then essential for the estimation of carrier concentration.

It should be mentioned that C_{gate} is usually approximated using the oxide capacitance (in our case that for 300 nm SiO_2), this is however based on the assumption of highly conductive semiconductor channel, e.g. the degenerately doped one in our manuscript. In the case of depleted channel, the small semiconductor capacitance start to determine the overall gate coupling. As a result, the mobility can be underestimated, which then leads to the overestimation of carrier concentration in depletion.

To avoid this situation, we adopt the simplified treatment in reference and estimate only those free carriers near band edge that contribute most to the measured conductance in MoS_2 and WSe_2, which is rational since they determine the essential application performance in electronic devices. According to Xiao et al.,[1] the free carrier mobility near band edge is not influenced by FE polarization. The free carrier concentration can be then approximated using the band edge mobility, written as $n_{free}=\sigma/\mu_{n,0}e$ for electrons (same for holes).

Here, we approached the band edge mobility in MoS_2 and WSe_2 by the extracted maximum carrier mobility from measured transfer curves under back gate modulation at each polarization. To be specific, for n-doped (p-doped) samples, $\mu_{n,0}$ ($\mu_{p,0}$) is approximated by the maximum mobility extracted at positive (negative) gate bias (V_{bg}=+30 or -30V) that raises the Fermi level close to the conduction (valance) band. The estimated free carrier concentrations in p and n doped MoS_2 and WSe_2 at different FE polarization voltages are supplied in Supplementary Figure 4. A summary of the tuned carrier concentration range is also given in Supplementary Table 1.

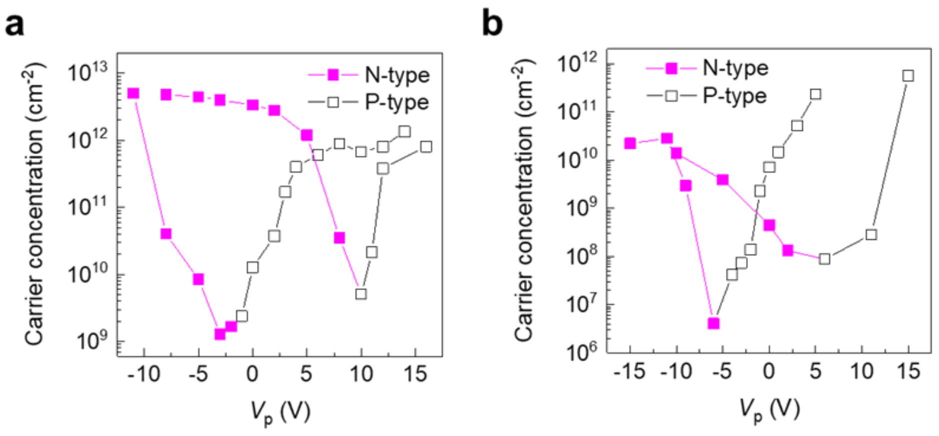

Supplementary Figure 4. FE polarization tuned doping polarity and carrier concentration. Free electron (purple) and hole (gray) concentration estimated from field effect measurements for **a** MoS_2 and **b** WSe_2 after FE polarization.

	Carrier Concentration (cm^{-2})			
	MoS_2 (e)	MoS_2 (h)	WSe_2 (e)	WSe_2 (h)
**Maximum**	$5x10^{12}$	10^{12}	$2x10^{10}$	$3x10^{11}$
**Minimum**	10^9	10^9	10^7	10^7

Supplementary Table 1. A summary of the extracted maximum and minimum free carrier concentration in FE-doped MoS_2 and WSe_2.

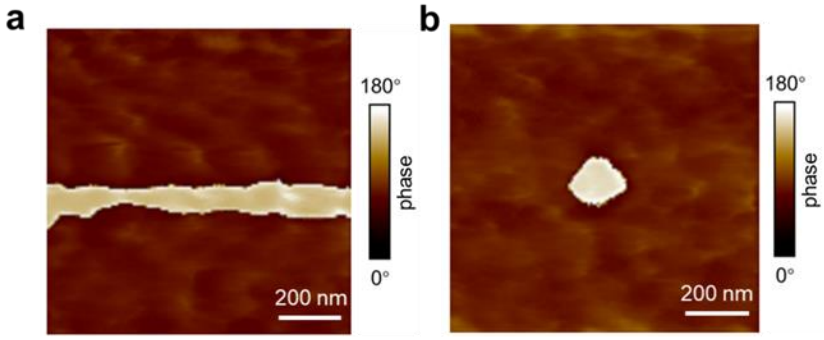

Supplementary Figure 5. Resolution limit of the ferroelectric patterning using scanning AFM tip. PFM phase image of P(VDF-TrFE) polarized by 10 V AFM tip using **a** line and **b** point scan mode, from what the FE domain size is estimated ~100-180 nm. In experiments, to define ferroelectric polarization pattern in device, scan resolutions of 256×256 or 512×512 are adopted for 10×10 μm^2 to 20×20 μm^2 device area, which means the fine spatial resolution of ~40 nm between adjacent line scans. The measured domain size is thus larger than the spatial resolution adopted in making FE pattern, therefore limited the switching region in forming **P**↑ and **P**↓ patterns.

Supplementary Note 2. Optimization of the pn junction configuration and characteristics.

We note that to improve the junction characteristic, one has to reduce the serial resistance to the pn junction. This is achieved by optimizing the position of pn junction defined in channel and by exploiting back gate modulation, which tuned the doping state in the channel area, therefore the serial resistance to pn junction area. Supplementary Figure 6 displays the characteristic of a pn junction defined in Dev-1 at the middle of the channel. In Supplementary Figure 7, the pn junction in Dev-1 is defined at different region, and the current characteristic under back-gate modulation is measured.

The diode formed with n-doped region span the whole channel area is found to exhibit robust rectification under gate modulation compared to the other case. This is attributed to the heavy electron doping in n-doped MoS_2 by FE polarization compared to gate modulation induced charge injection. On the other hand, the diode with p-doped channel can be dramatically modulated by the external gate bias, as discussed below.

In Supplementary Figure 7e, the junction current in forward bias manifests ambipolar characteristic, with p-type conductance appeared under negative V_g but n-type behavior appeared under positive V_g. This is because of the gate modulation effect to the p-doped channel. Due to the same reason, the pn diode transformed into i-n^+ Schottky diode under positive gate bias when hole carriers in p-doped channel are depleted. Under light illumination, the junction current under reverse bias starts to display n-type characteristics, as indicated in Supplementary Figure 7f, which is explained based on the n-type photo-doping effect in MoS_2 by minority carrier trapping.[2] As a results, the pn diode under illumination performed as a n-n^+ Schottky diode with significantly increased reverse current.

Based on the above observation, we therefore conclude that a robust pn diode can be formed by defining the pn junction near the contact terminal while keeping n-doped region span the whole channel.

To examine the diode characteristic, the *I-V* characteristic of defined pn diode is fitted using Equation 1 in main manuscript. An example of fitted junction current for Dev-1 is displayed in Supplementary Figure 8a, displaying an ideal factor n=3 and R_s=35 MΩ. In Supplementary Figure 8b, it is confirmed that the ideal factor can be optimized to ~1.4 using back gate modulation.

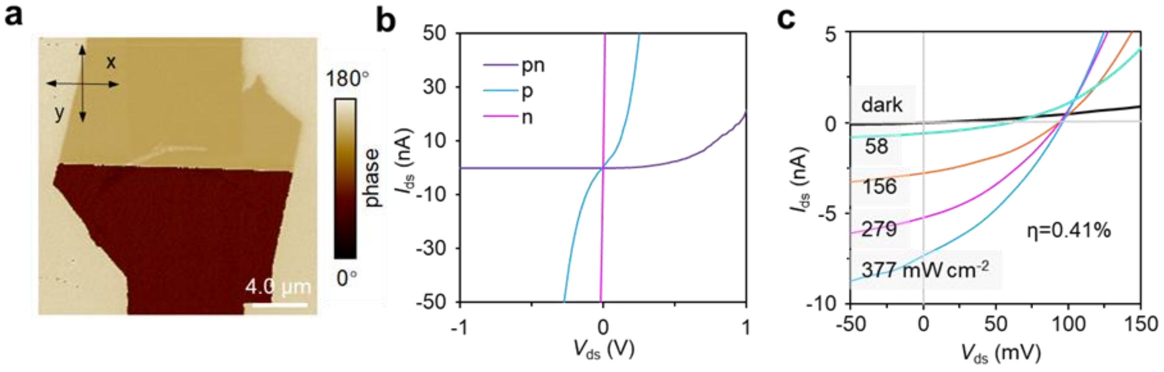

Supplementary Figure 6. Characteristic of pn junction defined on Dev-1. **a** PFM phase image of the FE polarization pattern for a lateral pn diode. **b** Comparison of the *I*-*V* characteristic of MoS_2 when configured into n, p doped states and pn patterned diode. **c** Photovoltaic effect of the pn diode under varied illumination intensities, from what an overall energy conversion efficiency of 0.41% is derived.

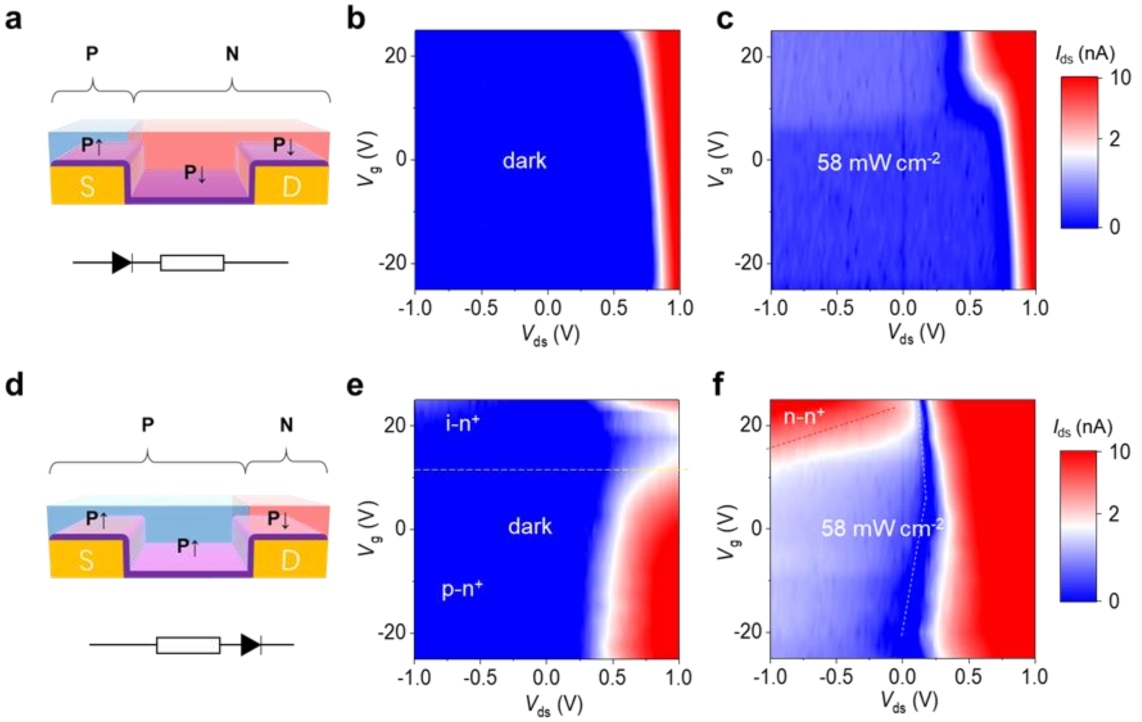

Supplementary Figure 7. Effect of the pn junction configuration to the diode characteristics in Dev-1. **a**, **d** illustration of the prepared pn diodes with the junction defined near the source or drain electrodes, and the measured color map of Si back-gate modulated junction current under **b**, **e** dark and **c**, **f** illuminated conditions.

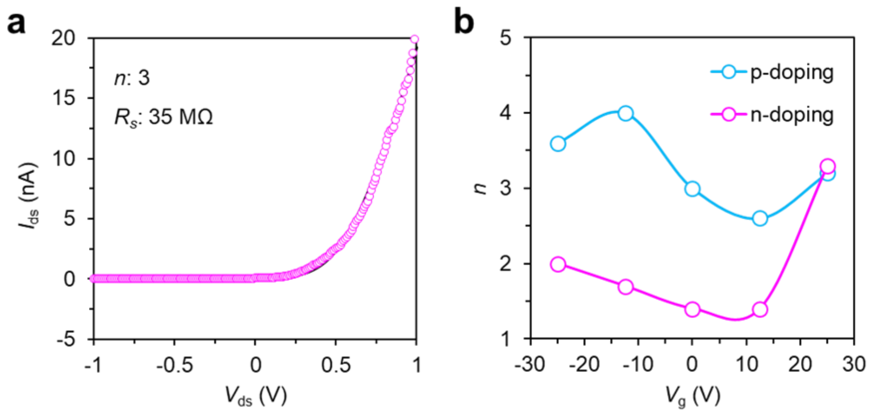

Supplementary Figure 8. Characteristic of the lateral pn diode and Si back-gate modulation. **a** Fitting to the junction current in the as-formed lateral pn junction in Dev-1 by $I=I_0\exp[e(V-IR_s)/nk_BT]$, where I_0 is the reverse saturation current, R_s is the serial resistance, n is the ideal factor. **b** Si back-gate modulation to the ideal factor n for the respective cases of n and p doped region spanning the channel area.

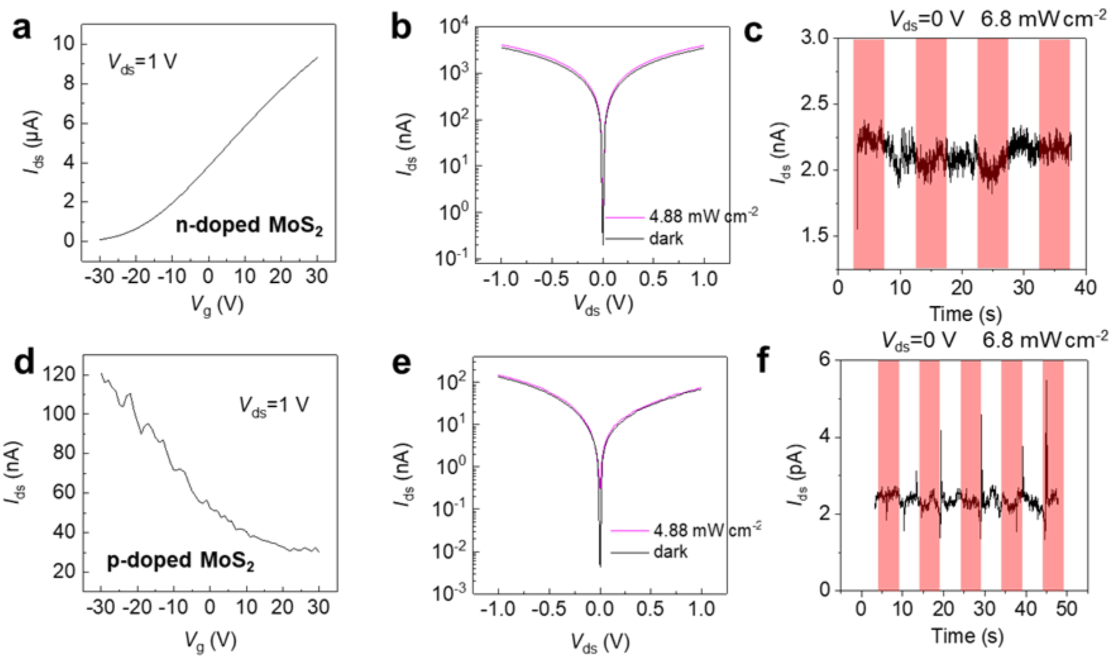

Supplementary Figure 9. Photocurrent characteristic of n and p-type doped MoS_2 in Dev-2 under complete ferroelectric polarization. **a**, **d** The measured transfer curves, **b**, **e** *I*-*V* curves, and **c**, **f** short-circuit photocurrent response for n, p doped MoS_2, respectively. The results confirm that the device without defining pn junctions displayed no self-driven photoresponse when the whole MoS_2 channel is polarized into n- or p-doped states.

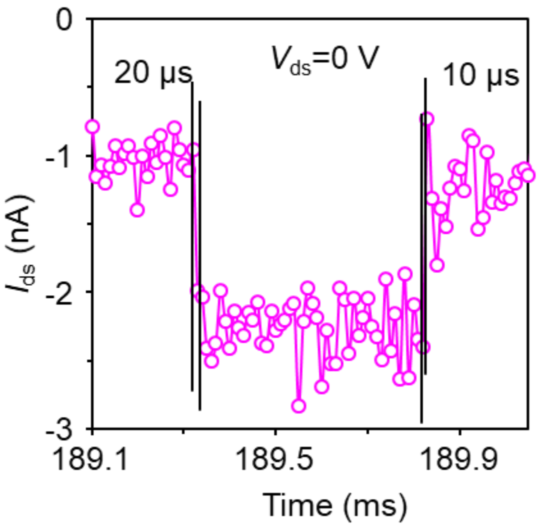

Supplementary Figure 10. Transient photoresponse of pn diode (Dev-2) in self-driven mode to fast switching light illumination at 365 nm. The response time is estimated ~10-20 μs. The appearance of significant noise in measurement is due to the sensitivity limit of adopted measurement unit (B1530, Agilent).

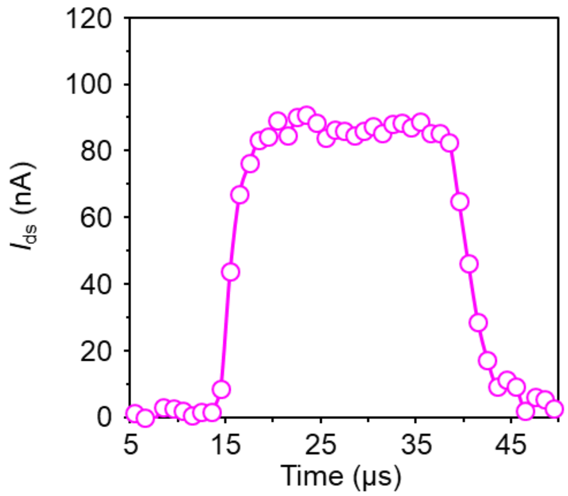

Supplementary Figure 11. Transient photoresponse of npn diode at V_{ds}=5 V to fast switching light illumination at 365 nm. The measurement is conducted using a sampling interval of 1 μs and the response time is estimated ~3-5 μs.

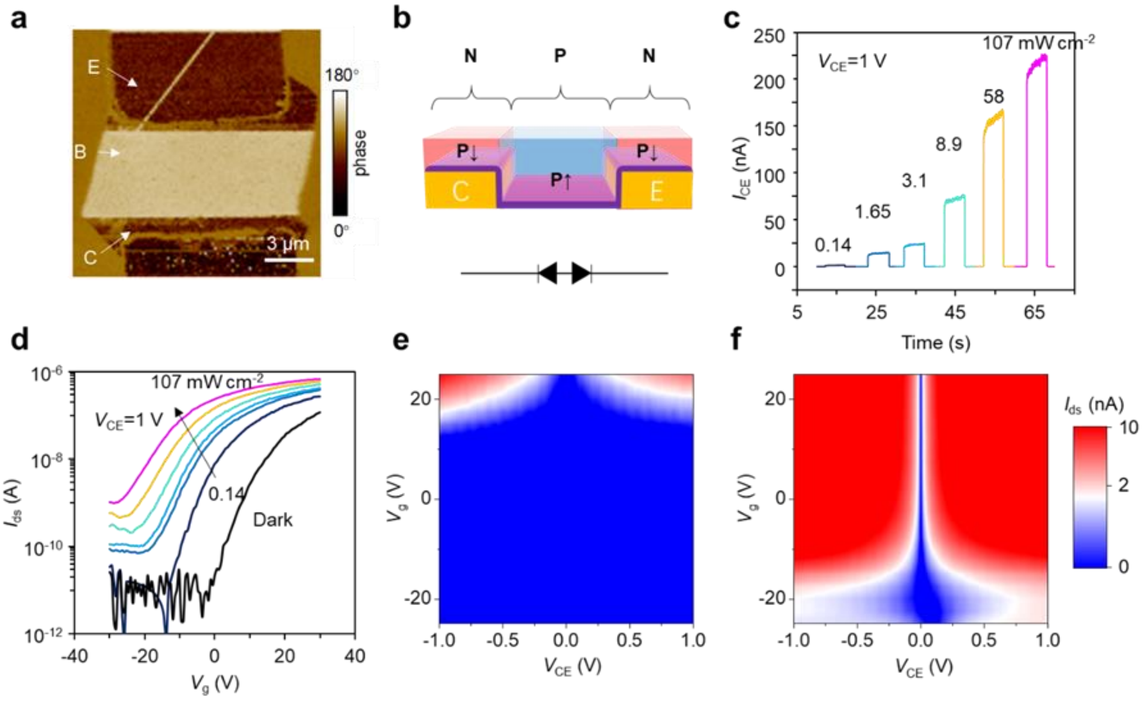

Supplementary Figure 12. Example of another npn bipolar transistor defined on 8 nm MoS_2. **a** PFM phase image of the FE polarization pattern for npn bipolar transistor, with emitter (E), base (B) and collector (C) defined respectively into n, p and n type. **b** An equivalent circuit of the npn transistor by two reversely connected pn diodes, yielding the low dark current of bipolar phototransistor. **c** Transient photoresponse and **d** transfer curves of the npn phototransistor under varied 532 nm light illumination conditions, with the intensity increased from dark to 107 mW cm^{-2}. **e** and **f** shows the color map of gate modulated current in npn bipolar transistor in the respective dark and illuminated conditions (58 mW cm^{-2}). The apparent negative shift of threshold voltage in illuminated transistor validates the increase of electron concentration in base by electron injection from the forward biased emitter to base (E-B) junction.

Table 2 | performance comparison of MoS_2 photodetectors in different device configuration					
Configuration	Materials	λ (nm)	Speed (s)	R ($A W^{-1}$)	Ref.
Photoconductor	MoS_2	561	20	880	3
	MoS_2	635	0.01	10	4
Photodiode	MoS_2/Lateral junction	575	0.75	0.308	5
Phototransistor	MoS_2 /Top gate	532	7×10^{-5}	0.57	6
	MoS_2/Vertical pn junction	635	0.01	7.8	7
Bipolar transistor	MoS_2-BP	1550	1.5×10^{-5}	0.153	8
	MoS_2 npn	532	2×10^{-5}	11.9	This work

Supplementary Table 2. A comparison to the photodetection performance of present npn bipolar transistor to other type MoS_2 photodetectors collected from literatures.[3-8]

Supplementary References

1. Xiao, Z., Song, J., Ferry, D. K., Ducharme, S. & Hong, X. Ferroelectric-Domain-Patterning-Controlled Schottky Junction State in Monolayer MoS_2. *Phys. Rev. Lett.* **118**, 236801 (2017).
2. Furchi, M. M., Polyushkin, D. K., Pospischil, A. & Mueller, T. Mechanisms of photoconductivity in atomically thin MoS_2. *Nano Lett.* **14**, 6165-6170 (2014).
3. Lopez-Sanchez, O., Lembke, D., Kayci, M., Radenovic, A. & Kis, A. Ultrasensitive photodetectors based on monolayer MoS_2. *Nat. Nanotechnol.* **8**, 497-501 (2013).
4. Kufer, D. & Konstantatos, G. Highly sensitive, encapsulated MoS_2 photodetector with gate controllable gain and speed. *Nano Lett.* **15**, 7307-7313 (2015).
5. Zhang, X. *et al.* Poly(4-styrenesulfonate)-induced sulfur vacancy self-healing strategy for monolayer MoS_2 homojunction photodiode. *Nat. Commun.* **8**, 15881 (2017).
6. Tsai, D. S. *et al.* Few-layer MoS_2 with high broadband photogain and fast optical switching for use in harsh environments. *ACS Nano* **7**, 3905-3911 (2013).
7. Huo, N. & Konstantatos, G. Ultrasensitive all-2D MoS_2 phototransistors enabled by an out-of-plane MoS_2 PN homojunction. *Nat. Commun.* **8**, 572 (2017).
8. Ye, L., Li, H., Chen, Z. & Xu, J. Near-infrared photodetector based on MoS_2/black phosphorus heterojunction. *ACS Photonics* **3**, 692-699 (2016).
